# Analysis of Mercury Concentration in Dietary Supplements Supporting Weight Loss and Health Risk Assessment

**DOI:** 10.3390/nu17111799

**Published:** 2025-05-26

**Authors:** Barbara Brodziak-Dopierała, Agnieszka Fischer, Zofia Wilk, Wojciech Roczniak, Magdalena Babuśka-Roczniak

**Affiliations:** 1 Department of Toxicology, Toxicological Analysis and Bioanalysis, Faculty of Pharmaceutical Science, Medical University of Silesia, 30 Ostrogórska Str., 41-200 Sosnowiec, Poland; afischer@sum.edu.pl (A.F.); s80145@365.sum.edu.pl (Z.W.); 2 Faculty of Medical Science, Jan Grodek State University in Sanok, 21 Mickiewicza Str., 38-500 Sanok, Poland; wroczniak@up-sanok.edu.pl (W.R.); mbabuska-roczniak@up-sanok.edu.pl (M.B.-R.)

**Keywords:** dietary supplements (DSs), slimming, mercury (Hg), health risk assessment

## Abstract

**Background:** Dietary supplements (DSs) are products classified as foodstuffs, but frequently wrongly identified as medicines. The aim of the study was to determine the mercury (Hg) content in DSs supporting weight loss. The analysis concerned DSs’ main active ingredient, form and manufacturer. The exposure to Hg resulting from the consumption of the supplements tested and the potential risk to health were also assessed. The results were compared to the permissible standards specified by the EU. **Materials and Methods:** The study used 47 preparations available on the Polish market. The studies were performed by means of an AMA 254 Hg analyzer using the atomic absorption spectrometry method with the amalgamation technique. **Results:** The Hg content in slimming DSs ranged from 0.12 to 46.27 µg/kg; the arithmetic mean was 5.80 µg/kg, whereas the median value was lower—2.44 µg/kg. The highest average Hg content (21.58 µg/kg) was found in supplements containing chlorella, followed by preparations containing white mulberry—10.98 µg/kg. The lowest Hg content was noted in preparations with L-carnitine (1.07 µg/kg). **Conclusions:** In none of the supplements tested did the amount of Hg exceed the permissible standard, indicating that their consumption in accordance with the manufacturer’s recommendations does not pose a risk to consumers’ health. To assess the risk of exposure, the THQ (Target Hazard Quotient) index was calculated and found to be higher than 1 for 11 DSs. Therefore, it can be concluded that there is a likely risk of side effects associated with the consumption of the DSs tested.

## 1. Introduction

According to WHO, in 2022 [1], approximately 16% of adults worldwide were obese. The prevalence of obesity has been observed to have more than doubled between 1990 and 2022 and quadrupled among adolescents. In 2022, 2.5 billion adults were overweight, and among them, 890 million were obese [1]. The prevalence of overweightness varies by region, from 31% in Southeast Asia and Africa to 67% in both Americas [2]. Obesity is also common in European countries. The prevalence of obesity ranged from 7.5 to 35.7%, being the highest in Spain (35.7%) and the United Kingdom (26%) [3], and was higher among men than women [4]. Global estimates suggest that almost 2.3 billion children and adults are living with overweightness and obesity. If current trends continue, 2.7 billion adults could be living with overweightness or obesity by 2025 [5]. Data released on World Obesity Day (March 4) predict that the total number of adults living with obesity will increase by more than 115% between 2010 and 2030, from 524 million to 1.13 billion. By 2025, the global prevalence of obesity is predicted to reach 18% in men and exceed 21% in women [6].The consequences of obesity are associated with the development of many diseases and are becoming an increasing challenge for both health and social policy [7]. Diabetes, hypertension, heart complications, depression and dyslipidemia were the most frequently reported comorbidities. Obesity also significantly affected the psychosocial sphere and the health-related quality of life (HRQoL) index [3].

A significant interaction was observed between high genetic risk of obesity and unhealthy lifestyle. Despite the high genetic risk of obesity, individuals can prevent comorbidities by following a healthy lifestyle and maintaining a normal body weight. A healthy lifestyle should be promoted regardless of genetic predispositions [8].

DSs are defined by European law as “foodstuffs intended to supplement the normal diet and which are a concentrated source of nutrients or other substances with a nutritional or physiological effect, single or combined, marketed in dose form…”. They usually contain vitamins, amino acids, minerals, herbs and other substances or their components. DSs are very often confused with over-the-counter medicinal products [9,10,11]. The DS market is very large, probably reaching USD 327.4 billion by 2030 [12]. DSs are advertised to fight obesity. The slimming supplements are the most frequently searched dietary supplements on the Internet [13]. In the USA, over 50% of adults have declared using DSs [14,15]. In Poland, 30–78% of teenagers and adults have reported taking supplements [16]. Weight loss supplements are usually multi-ingredient preparations. Their effect on the body is difficult to determine due to complexity of the recipes, the quantitative diversity of active ingredients and the dosing periods [12]. DS and unconventional weight loss therapies have limited effectiveness, as confirmed by numerous studies [17].

Research indicates that taking weight loss supplements may create the illusion of protection against weight gain and attenuate dietary self-control. After taking slimming supplements, the consumers may feel overly optimistic about losing weight and allow themselves to overeat [18].

Metals, including mercury (Hg), are common contaminants in food and DSs due to their ability to penetrate from soil, water and air. Hg is an unavoidable environmental pollutant and can pose a serious threat to human health. Many in vivo and in vitro studies have demonstrated that the disruption of redox homeostasis and microtube assembly is mainly responsible for mercurial toxicity leading to adverse health outcomes [19]. Mercury can bind sulfhydryl groups in cells, which causes nonspecific cell damage and even cell death, inhibition of microtubule formation, enzyme inhibition, oxidative stress, inhibition of protein and DNA synthesis, and autoimmune reactions [20]. Many studies have indicated numerous adverse effects of Hg, mainly including hepatotoxicity, nephrotoxicity and heart problems [21,22,23,24,25,26,27,28,29,30]. Heavy metals, including Hg, can affect the physiological and biochemical functions of plants, which in turn affects photosynthesis by reducing chlorophyll production, impairing enzymatic activity and hindering the absorption of nutrients. In addition, heavy metals can cause oxidative stress in plants. They can produce reactive oxygen species, which can damage cellular structures and disrupt metabolic functions [31]. DSs, especially those supporting weight loss, are usually multi-ingredient preparations, containing even 10 or more ingredients. With such a complex composition, it is difficult to determine their effect on the body. According to the United States Anti-Doping Agency, 20–25% of all DSs contain banned substances, although manufacturers do not include this information in the product composition [32].

Commission Regulation (EC) No. 2023/915 of 25 April 2023 amending Regulation (EC) No. 1881/2006 established maximum permissible levels for certain contaminants in foodstuffs, specifying the ML (maximum level) of contaminants, including Hg, to be 0.10 mg/kg in food supplements [33].

The study objective was to determine the Hg content in weight loss-supporting DSs available on the Polish market. Hg concentration was determined in products from different manufacturers containing different active ingredients. DSs were divided according to the type of the main ingredient present in the product. The obtained Hg concentration values in the tested DSs were compared with the ML of the Regulation of the European Commission [28] to determine whether the intake of DS poses a risk to consumers due to the level of Hg. The estimated daily intake (EDI), the estimated weekly intake (EWI), the % tolerable weekly intake (%TWI) and the Target Hazard Quotient (THQ) were calculated to assess the exposure to Hg resulting from the consumption of the DS tested.

## 2. Materials and Methods

### 2.1. Materials

The study material consisted of 47 DSs used to support weight loss, from various manufacturers, obtained from stationary pharmacies in Poland and online stores from 2023 to 2024. Slimming DSs differed in terms of qualitative composition. They included preparations with one, a few or even several ingredients. The main active and other ingredients and their manufacturers are presented in Table 1. They were grouped according to the main component and form. The main components of the DSs were spirulina, white mulberry, young barley, Malabar tamarind, chlorella, green coffee, l-carnitine, African mango and Indian barberry. Most of the products were in the form of tablets (N = 30), with the others in the form of powders within capsules (N = 17). The characteristics of the DSs used for the study are presented in Table 1.

The DS samples were first crushed using a porcelain mortar to obtain a uniform powder. Then, approximately 50 mg of each sample was weighed out using an analytical balance (analytical balance, OHAUS, Parsippany, NJ, USA), preparing 3 weighed portions.

### 2.2. Analytical Method

The total Hg content of the test samples was determined using atomic absorption spectrometry (AAS) by an AMA 254 analyzer (Altec, Prague, Czech Republic). This method uses the amalgamation technique, allowing for the determination of total Hg regardless of its form, which is facilitated by the pyrolytic mineralization process inside the spectrometer, as it occurs without the need for prior sample preparation [29]. The following measuring conditions were used: wavelength 253.65 nm, carrier gas—oxygen (O_2_ purity ≥ 99.5%), inlet pressure 200–250 kPa. The time of each analytical step was as follows [seconds]: drying 120, decomposition 140, measurement 60. The lower detection limit (LOD) was 0.01 ng. Prior to each measurement, the apparatus was cleaned with air and distilled water in accordance with the analytical procedure [34]. The original factory calibration was valid for instrument calibration. The certified reference material (CRM) was used to ensure quality control of the method. Mixed Polish Herb INCT-MPH-2 (Institute of Nuclear Chemistry and Technology, Warsaw, Poland) was used, with the following result from six analyses: 0.018 ± 0.002 mg/kg, recovery 92%. The device was controlled using an external PC running Windows^®^, which had advanced software enabling, among others, creation of calibration curves, statistical analysis of results and control of the ongoing process, with current signal display. Prior to each analysis, the device was cleaned in air and then in deionized water (Elix Essential Water Purification, Merck, Darmstadt, Germany). From each DS preparation, 3 samples were prepared and tested once on the AMA 254 apparatus. Three measurements of each sample were performed and the arithmetic mean was calculated from them.

### 2.3. Health Risk Assessment

To assess the exposure to Hg resulting from the consumption of DSs, the following were calculated: the estimated daily intake (EDI) and the estimated weekly intake (EWI). The daily intake of DSs ranged from 1 to 20 tablets/powders within capsules according to the manufacturer’s recommendations placed on the packaging. The estimated daily intake was calculated by multiplying the concentration of Hg in the analyzed product and the portion of the analyzed product consumed daily. In order to relate the obtained Hg contents in DSs to the TWI (tolerable weekly intake) values, the %TWI was calculated. TWI is 4 µg/kg body weight in the case of inorganic compounds [35,36]. For the organic compounds, EFSA has revised the value for methylmercury, lowering the previous value to 1.3 µg/kg b.w. [37]. Applying these values to an average human of 70 kg, the TWI for inorganic mercury is 280 µg and the TWI for methylmercury is 91 µg.

%TWI_inorganic mercury_ = EWI/280 × 100%

%TWI_methylmercury_ = EWI/91 × 100%

The Target Hazard Quotient (THQ) index was also calculated to analyze the potential non-cancer effects of metals present in food.

The THQ was calculated according to the following equation [38,39,40,41,42]:

THQ = ADD/RfD

where

ADD—average potential daily dose,

RfD reference dose value for Hg = 0.0001 mg/kg/day [43,44].

The ADD value was calculated from the quotient of EDI and body weight; the average value was assumed to be approximately 70 kg.

The THQ was interpreted as follows:

THQ < 1—the risk of adverse health effects in the population is unlikely, and is associated with a low non-cancerogenic risk;

THQ ≥ 1—the risk of adverse effects in the population is not excluded, and the hazard is probable.

### 2.4. Statistical Analysis

The obtained results were statistically analyzed using Microsoft Excel 2007 and Statistic aver. 13.3 pl for the Windows operating system [45].

The following were determined: arithmetic mean—AM, standard deviation—SD, median—Me, first quartile—Q_1_, third quartile—Q_3_, coefficient of variation—CV. The distribution of variables was evaluated using the Shapiro–Wilk test. Nonparametric tests were used to compare statistical variability between the tested sample groups: Mann–Whitney U (for 2 groups) and Kruskal–Wallis ANOVA ranks (for more groups). The statistically significant probability level was *p* < 0.05.

## 3. Results

The mean Hg content in all the slimming DS samples was 5.80 µg/kg; the median value was lower—2.44 µg/kg. The obtained values were characterized by a high coefficient of variation (146%), suggesting a large variation in mercury content. The minimum content was 0.12 and the maximum was 46.27 µg/kg.

The DSs tested were divided, taking into account the main largest component. It was difficult to obtain preparations with a similar composition due to a great variety of ingredients among the available slimming DSs. In the DSs tested, 19 different main ingredients were distinguished. Samples with a quantity of ≥3 were used for statistical analysis. The group of 11 ingredients, which had one or two supplements, was not included in the statistical analysis. The statistical analysis for individual DS groups is included in Table 2. No statistically significant differences were found in the main ingredient in the respective DSs tested. The highest average Hg content (21.58 µg/kg) was found in supplements containing chlorella, followed by preparations containing white mulberry—10.98 µg/kg (almost twice as low). High Hg content translated into high values of the coefficient of variation (108 and 92%) for preparations containing chlorella and white mulberry, respectively. The amount of Hg was found to be close to the average value for all DSs in preparations containing spirulina (6.13 µg/kg), young barley (5.09 µg/kg) and Malabar tamarind (3.99 µg/kg). An amount of Hg below the average value was observed in supplements with green coffee (2.10 µg/kg) and African mango (1.57 µg/kg). The lowest Hg content was noted in preparations with L-carnitine (1.07 µg/kg).

**Figure 1:**
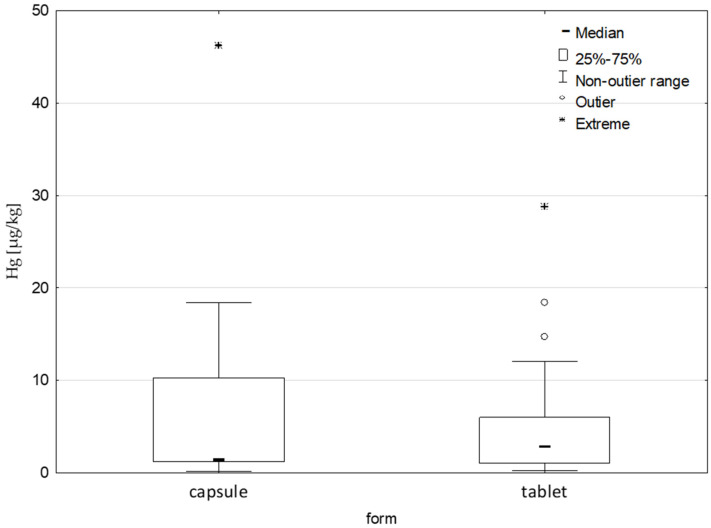
Hg content [µg/kg] in the tested weight loss-supporting DSs depending on the form of the supplement, *p* > 0.05.

The DSs tested were in the form of tablets (N = 30) or powders within capsules (N = 17). The arithmetic mean value was higher in the case of powders within capsules (7.15 µg/kg) than tablets (5.03 µg/kg)–Table 3. There were no statistically significant differences between the form of the supplement and the Hg content—Figure 1.

**Figure 2:**
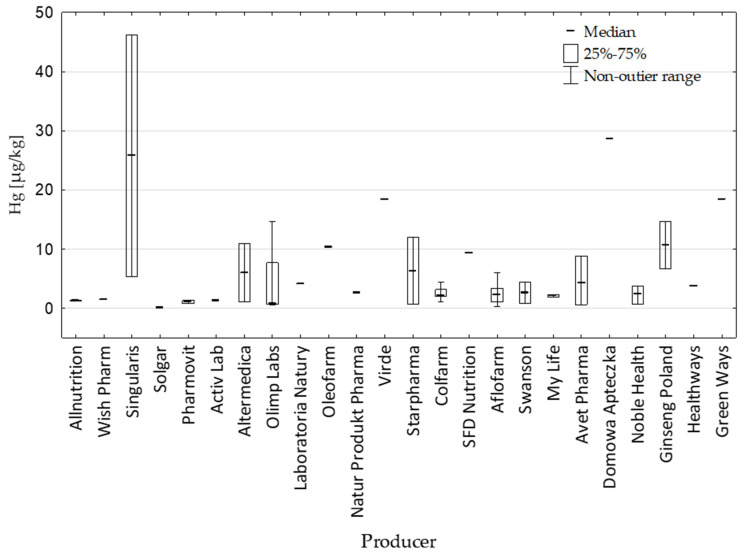
Hg content [µg/kg] in slimming DSs depending on the manufacturer.

The slimming dietary supplements used in these studies were manufactured by 24 different companies. The largest number of supplements (N = 5) came from Aflofarm and Colfarm and Olimp Labs (N = 4). The average Hg concentrations in DSs from these companies were similar, ranging within 2.55–4.17 µg/kg—Figure 2.

Based on the Hg content in the tested DSs, the hypothetical amount of Hg consumed with these preparations was calculated, taking into account the recommended dosage and different times of consumption—EDI, EWI. The analysis of health risk also included the % TWI for methylmercury and inorganic mercury, their sum and THQ—the results are presented in Table 4.

The EDI index values were in the range of 0.0001–0.1212 µg. The two highest values (0.1212 and 0.0763 µg) were found for supplements no. 47 and 3 containing chlorella, then for the preparation with white mulberry (0.0603 µg), no. 23, and for three supplements with spirulina (0.0201–0.0439 µg), no. 34, 37 and 38. The lowest EDI value was noted for the preparation containing *Cola nitida* extract (0.0001 µg)—no. 11. The average value for all the DSs tested was 0.011 µg. The differences between the lowest and highest values were very significant (approximately 1800 times). The EWI indices were the lowest and highest in the same supplements as for EDI.

Next, the TWI% was determined for both forms of Hg (inorganic mercury and methylmercury) found in food. None of the dietary supplements tested exceeded this value. The TWI% values ranged from 0.0009 to 1.23%.

**Figure 3:**
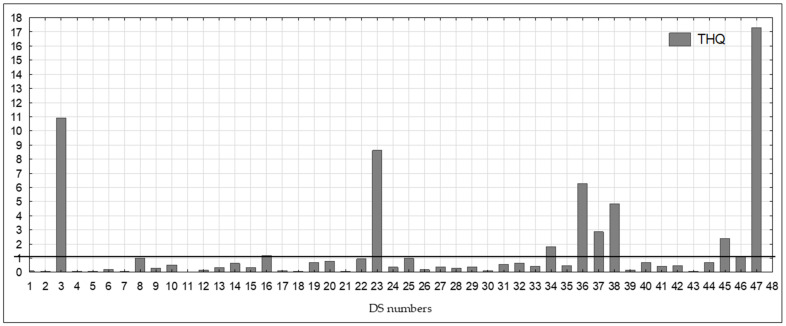
Calculated THQ values for the tested DS.

Based on the calculated THQ values, it could be concluded that the risk of adverse health effects in the population is unlikely for the majority of the slimming DSs tested (THQ values < 1). The THQ values in the 36 DS samples studied were in the range of 0.01–0.98. However, 11 DS samples, including preparations containing spirulina (N = 4)—no. 34, 36, 37, 38, chlorella (N = 2)—no. 3, 47, white mulberry extract (N = 2)—no. 23, 45, African mango (N = 1)—no. 16, Malabar tamarind (N = 1) and green tea extract (N = 1)—no. 46, showed THQ values ≥ 1 (range 1–17.31) (Figure 3).

## 4. Discussion

Consumers choose dietary supplements under the influence of advertisements that suggest an easy-to-achieve goal, e.g., weight loss, healthy and thick hair, strong nails or beautiful skin. Stylized visualizations encourage people to buy dietary supplements. In addition, there is a belief that natural, i.e., plant-based products, unlike synthetic ones, cannot do any harm to health. However, numerous studies indicate adverse effects of dietary supplements, including toxic ones [46,47].

In pharmacies, shelves are labelled with such inscriptions as “slimming products”, which gives credibility and indicates the clinical effectiveness of plant-based raw materials, including caffeine, capsaicin and other compounds. However, there is no or little evidence of their action [12,17,19,48]. It is important to make sure that plant raw materials do not contain such contaminants as heavy metals, pesticides or polycyclic aromatic compounds. Contamination can also be caused by reactions during processing, production and storage.

Uncontrolled consumption of dietary supplements makes it impossible to determine whether their side effects depend on improper dosage and/or time of administration [12,15,25,26]. Research has shown that people usually decide to use supplements on their own, which was declared by about 70% of students [49]. Very few of those taking DSs consulted with a doctor or a dietician about their intake (about 4% of respondents) [50]. It should also be noted that various slimming products (with different trade names) may contain the same ingredients, and their concentration in the body may be dangerous. The long-term effects of using supplements are difficult to predict and can only be thoroughly examined after a year of marketing [13,51]. However, the dynamics of changes in DSs are very high, new products are constantly appearing and older ones are being withdrawn from sale. The lack of reliable data on the composition, action and safety of DSs justifies the need to conduct research into this group of products and to educate health care professionals and consumers on their safe use. Many previous studies have shown that the Hg content in herbal DSs is higher than in vitamin–mineral preparations, e.g., 3.9 v 1.8 µg/kg [51,52,53]. The use of plant DSs was found to be contraindicated mainly in gastrointestinal diseases (16.4%), neurological diseases (14.5%) and kidney/urinary diseases (12.5%). The largest number of documented contraindications was found for linseed, echinacea and yohimbine [54].

Puścion-Jakubik et al. [40] analyzed slimming DSs from Poland. The average concentration of Hg was 3.08 µg/kg and Me = 1.78 µg/kg. These values were lower compared to those obtained in the current study (5.80 µg/kg and 2.44 µg/kg, respectively). The differences may result from the different group of supplements that was used in their study, and the DSs and the main ingredient they contained were not specified. The highest level of Hg in the study by Puścion-Jakubik et al. [40] was found in the group of supplements regulating glucose levels—23.97 µg/kg—and these were products containing extracts of *Gymnema sylvestre* and *Trigonella foenum graceum*. In the current study, only one product contained extracts of *Gymnema sylvestre*, but it was not the main ingredient. It is interesting, however, that in our study, products containing white mulberry leaf extract (N = 6) also had a high Hg content of 10.98 µg/kg (Me = 8.11 µg/kg).

In our study, the highest THg content was found in DSs containing *Chlorella vulgaris*—21.58 µg/kg (Me = 18.36 µg/kg). Similar results were obtained in previous studies from 2018 [55], for a product containing *Chlorella pyrenoidosa*.

Chlorella belongs to the green algae class, inhabiting moist and aquatic environments, both fresh and salty. It is a common food product in Japan and China. Studies have shown that algae can effectively absorb and accumulate heavy metals from polluted areas in which they grow [55]. This feature of algae can be used to remove contaminants from water and industrial wastewater [56]. Chlorella in DSs is a source of vitamins, free fatty acids and micro and macro elements, removes toxins, has an antioxidant effect and supports the immune system. Large doses are recommended (3–20 tablets per day). High Hg content, high dosage and long period of use result in high EDI, EWI, EMI, annual intake and THQ values.

Our previous study [51] indicated a higher Hg content in medicines (3.9 µg/kg) than in DSs (2.7 µg/kg), but these differences were not statistically significant. In the case of different types of DS designed to improve the condition of skin, hair and nails; vitamins; minerals; probiotics; specialties for women and weight loss, the highest Hg content was in weight loss DSs at 5.7 µg/kg [51]. In the current study, the Hg content in slimming and cleansing DSs was at a similar level of 5.8 µg/kg. Ćwieląg-Drabek et al. [39], in a study on DSs produced from terrestrial plants or microalgae, showed that approximately 30% of the DSs tested were contaminated with Hg. A higher content of Hg was determined in DSs from terrestrial plants—5 µg/kg (maximum 28 µg/kg)—than in DSs containing microalgae—3 µg/kg (maximum 17 µg/kg) [57]. In our study, the average Hg content in the DSs tested was very similar—5.8 µg/kg. However, differences were found in the case of DSs containing spirulina and chlorella. In the study by Ćwieląg-Drabek et al. [39], the amount of Hg in these DSs was lower than in plant DSs. In our study, the amount of Hg was the highest in products containing chlorella (21.58 µg/kg) and above the arithmetic mean in supplements containing spirulina (6.13 µg/kg). In DSs containing microalgae, the average Hg content was 13.86 µg/kg, being over 4 times higher than in the study by Ćwieląg-Drabek et al. [39].

Studies on Hg in medicines and DSs were also conducted by Kowalski and Frankowski [57]. They mainly analyzed the Hg content in prescription and over-the-counter medicines available on the Polish market, as well as in DSs containing micro- and macroelements, DSs containing vitamins and DSs improving the condition of the skin, hair and nails. The average Hg concentration in these supplements was 5.5 µg/kg [57], which was slightly lower than in the current study (5.8 µg/kg). The highest Hg content in DSs was in the vitamin supplement group, with a median value of approximately 7.5 µg/kg [57].

Further research by Socha et al. [58] on the Hg content in DSs confirmed the presence of Hg in the average amount of 5.36 µg/kg, in the range of 0.1–47.99 µg/kg. These values are very similar to those obtained in the current study (AM = 5.8 µg/kg; Min–Max = 0.12–46.27 µg/kg). Socha et al. [58] noted the highest Hg values in DSs affecting the urinary system—9.98 µg/kg—and the lowest in strengthening-stimulating preparations—2.37 µg/kg. The DS groups studied by us were not specified in the study by Socha et al. [58].

In the study performed by Uslu et al. [59], no Hg was found in multivitamin/mineral effervescent tablet supplements. The concentration of this metal was below the detection limit.

Kim [60] analyzed the content of selected elements, including Hg, in DSs available on the Korean market. The average Hg content was 10 µg/kg. The highest Hg level was found in preparations containing cartilage (60 µg/kg). Thus, the average daily intake of Hg and Cd was approximately 0.1–0.2 µg/kg, and both elements constituted less than 0.4% TWI [60].

The study on DS containing young green barley (*Hordeum vulgare* L.) [61] included 31 samples in the form of tablets, powders and juices, and no Hg was detected in only 1 sample. The highest Hg content was in powders—19.1 µg/kg—and then in tablets—15.3 µg/kg. The lowest was in juices—0.7 µg/kg. Interestingly, the highest Hg content (760 µg/kg) was observed in a sample from organic farming. In the study by Sadowska-Rociek et al. [61], three tested preparations exceeded the permissible Hg content established for DS (100 µg/kg).

In our research on DS containing young green barley, the average Hg content was lower, amounting to 5.09 µg/kg, and the products were in the form of tablets [62]. Beetroot-based DSs in the form of powders contained the largest number of harmful elements [42], similarly to the findings by Sadowska-Rociek et al. [61], who concluded that in most cases, DSs provided less minerals than a 100 g portion of fresh beets.

However, that study did not include Hg. Based on the obtained results and the works cited [39,40,51,52,61,62], it can be stated that in most cases DSs do not pose a threat to consumers’ health. However, in almost every study there are samples in which the concentration of Hg exceeds the permissible standards.

The use of DSs in accordance with the recommended dose constitutes 0.0009% TWI for sample no. 11 (containing extract from the seeds of *Cola nitida*) and 1.23% TWI for sample no. 47 (containing chlorella). The average value for all tested DS was 0.11% TWI. It should be noted here that this applies to both inorganic mercury and methylmercury. These values indicate that the DSs used are safe and do not pose a health risk in the case of Hg. In the study by Socha et al. [58], the %TWI values were in the range of 0.06–0.25%.

Maximum level is the level required in order to be allowed to export and sell the product, to protect the health of European Union (EU) citizens.

Sadowska-Rociek et al. [61] also showed that the TWI values for Hg were lower than the levels established by EFSA. This indicates that there is no significant risk associated with the consumption of the tested products in the recommended dose. In the case of DSs containing ingredients of plant origin in the Puścion-Jakubik study [40], the lowest TWI values were 0.001% and the highest 1.143%, with most samples having values ranging from 0.001 to 0.030%, thus being lower than in the current study. Our study showed that the Hg content in all tested DSs was below 100 µg/kg and did not exceed the maximum level set by the EU. The highest determined Hg value was half the maximum level. The RfD value according to the EPA is used to calculate the THQ value [44]. The THQ index seems to be a more sensitive indicator. Based on the calculated THQ values (THQ < 1), it was found that the consumption of most of the tested DSs and the risk of adverse health effects in the population are unlikely. For 11 samples of the tested DS, THQ values were ≥ 1. This referred to the preparations containing spirulina (N = 4), chlorella (N = 2), white mulberry extract (N = 2), African mango (N = 1), *Garcinia cambogia* (N = 1) and green tea extract (N = 1). THQ values ranged from 1 to 17.31—Figure 3. THQ values ≥ 1 indicate a possible risk of adverse health effects.

In the case of toxins that have the ability to accumulate in the body, the time of exposure is an important factor. Therefore, the THQ index is more sensitive compared to the TWI. In the studies by Brzezińska-Rojek et al. [63], the TWI value for cadmium in beetroot-based dietary supplements was exceeded. The TWI and THQ indices are most important for particularly sensitive populations, e.g., infants [64].

The limitations of the study are too few DS samples, especially preparations containing the following main active ingredients: red tea, bitter orange, prickly pear and green tea. Also, there is no information about other toxic metals present in DSs. The strength of the study is that it provides information about the Hg content of DSs. DSs are widely advertised as helpful and harmless, with little talk about potential harmful effects and possible interactions.

However, it should be remembered that dietary supplements are only one of many sources of Hg in the human body. Taking into consideration the cumulative amount of Hg from various sources (food, especially of marine origin, water, air), it may turn out that the amount of Hg and other metals is significant and may have a toxic effect on the body. Assuming that the consumption of slimming DSs is one of many sources of Hg, it should be considered whether the use of this type of product is justified.

## 5. Conclusions

The Hg content in all the analyzed weight loss dietary supplements was in the range of 0.12–46.27 µg/kg, and the arithmetic mean was 5.26 µg/kg. No statistically significant differences were found in the Hg concentration depending on the type of the main ingredient or the manufacturer.

The arithmetic mean Hg content was higher in dietary supplements in the form of powders within capsules (6.83 µg/kg) compared to tablets (4.37 µg/kg), but these were not statistically significant differences.

It should be mentioned that the amount of Hg in DSs determined in the study did not exceed the maximum permissible concentration in foodstuffs specified by the EU Commission Regulation. The TWI values for inorganic mercury and methylmercury compounds and their sum were also not exceeded; they did not pose a threat to consumer health. Mercury in DSs has little influence on the TWI value. All 47 DSs were tested; of these, 11 had a THQ value above 1 (ranging from 1 to 17.31). The study indicated that the THQ index is a more sensitive indicator as compared to TWI. DSs should be subject to continuous monitoring for toxic metal content before being released for sale. When advertising DSs, attention should be paid to possible overdoses and interactions with medications. In order to ensure consumer safety, more rigorous DS control measures should be implemented. In further research on DSs, it would be necessary to include products in different forms for consumption (e.g., powders, tablets, juices) containing the same main ingredient, since studies show that powders have the highest Hg content.

## Figures and Tables

**Table 1:** Summary of the tested DSs (dietary supplements) supporting weight loss, their composition, manufacturer, form and Hg content [µg/kg] (AM—arithmetic mean, SD—standard deviation).

No.	Main Active Ingredient	Other Ingredients	Manufacturer	Form	Weight [mg]	AM	SD
1	Indian barberry root extract (*Berberis aristata*)	-	Allnutrition (Opole, Poland)	powder within the capsule	500	1.32	0.05
2	Indian barberry root extract (*Berberis aristata*)	-	Wish Pharma (Cracow, Poland)	powder within the capsule	400	1.39	0.06
3	Chlorella (*Chlorella vulgaris*)	-	Solgar (Manhattan, NY, USA)	powder within the capsule	550	46.27	1.25
4	Chlorella (*Chlorella vulgaris*)	-	Singularis (Lewes, DE, USA)	powder within the capsule	1560	0.12	0.03
5	Red tea leaf extract (*Camellia sinensis* O.)	Common bean seed extract, L-carnitine tartrate, Cayenne pepper fruit extract	Starpharma (Warsaw, Poland)	tablet	400	0.67	0.12
6	Common bean seed extract (*Phaseolus vulgaris* L.)	Green tea extract, L-carnitine tartrate, extract of Indian barberry root, hake, guarana, coffee, ginger	Colfarm (Mielec, Poland)	tablet	750	2.04	0.04
7	Malabar tamarind fruit extract (*Garcinia Cambogia*)	-	Pharmovit (Płock, Poland)	powder within the capsule	400	0.80	0.04
8	Malabar tamarind fruit extract (*Garcinia Cambogia*)	-	SFD Nutrition (Opole, Poland)	tablet	750	9.29	0.61
9	Malabar tamarind fruit extract (*Garcinia Cambogia*)	Prickly pear fruit powder, extracts from the leaves of the hollyhock, spirulina, chromium	Aflofarm (Pabianice, Poland)	tablet	600	3.34	0.33
10	Bitter orange fruit extract (*Citrus aurantium)*	Green tea extract, apple cider vinegar, brown algae extract complex, grape seed extract, L-carnitine tartrate	Aflofarm (Pabianice, Poland)	tablet	600	5.98	0.40
11	Cola seed extract (*Cola nitida*)	Extract from fruit of cayenne pepper, leaves of Paraguayan holly, fruit of Malabrian tamarind	Aflofarm (Pabianice, Poland)	tablet	200	0.22	0.08
12	Hydroxycitric acid	Choline, inositol, L-methionine, chromium, apple cider vinegar powder, grapefruit powder, lecithin, taurine	Swanso (Fargo, ND, USA)	tablet	700	0.80	0.09
13	L-carnitine tartrate, bitter orange fruit extract (*Citrus aurantium*)	Green tea leaf extract, Malabar tamarind fruit, L-tyrosine, Cayenne pepper, black pepper fruit, chromium	Activlab (Kłaj, Poland)	powder within the capsule	620	1.28	0.08
14	L-carnitine tartrate	L-ornithine, L-arginine, chromium	Olimp Labs (Dębica, Poland)	tablet	1900	0.77	0.07
15	L-carnitine tartrate	Bitter orange fruit extract, green tea leaves, Malabar tamarind fruit, Cayenne pepper, black pepper fruit, N-acetyl L-tyrosine, chromium	Allnutrition (Opole, Poland)	powder within the capsule	700	1.16	0.13
16	African mango seed extract (*Irvingia gabonensis*)	-	My Life (Gniezno, Poland)	tablet	1200	2.31	0.32
17	African mango seed extract (*Irvingia gabonensis*)	-	Pharmovit (Płock, Poland)	powder within the capsule	500	1.33	0.03
18	African mango seed extract (*Irvingia gabonensis*)	-	Alter Medica (Żywiec, Poland)	powder within the capsule	540	1.09	0.11
19	Young barley (*Hordeum vulgare* L.)	-	Alter Medica (Żywiec, Poland)	powder within the capsule	220	10.89	0.05
20	Young barley (*Hordeum vulgare* L.)	-	Singularis (Lewes, DE, USA)	powder within the capsule	500	5.34	0.08
21	Young barley *Hordeum vulgare* L.)	Green tea, bitter orange, spirulina, chromium	Colfarm (Mielec, Poland)	tablet	250	1.13	0.04
22	Young barley (*Hordeum vulgare* L.)	Bitter orange, chromium, D-biotin.	Avet Pharma (Warsaw, Poland)	tablet	375	8.79	0.23
23	White mulberry leaf extract (*Morus alba* L.)	Cinnamon bark, chromium	Domowa Apteczka (Grodziska Mazowiecki, Poland)	tablet	700	28.74	0.58
24	White mulberry leaf extract (*Morus alba* L.)	Extract from cinnamon bark, chromium	Avet Pharma (Warsaw, Poland)	tablet	600	4.21	0.38
25	White mulberry leaf extract (*Morus alba* L.)	-	Olimp Labs (Dębica, Poland)	powder within the capsule	470	14.63	1.40
26	White mulberry leaf extract (*Morus alba* L.)	-	Colfarm (Mielec, Poland)	tablet	600	2.15	0.24
27	White mulberry leaf extract (*Morus alba* L.)	Extract from cinnamon bark, chromium	Laboratoria Natury (Lublin, Poland)	powder within the capsule	310	4.16	0.19
28	Prickly pear fruit powder (*Opuntia ficus-indica*)	Extract from leaves of *Gymnema sylvestre*, extract from bitter orange fruit	Aflofarm (Pabianice, Poland)	tablet	966	1.03	0.15
29	Yerba mate leaf extract (*Ilex Paraguariensis A.*)	Green tea extract, white bean extract, L-carnitine, spirulina, chromium	Aflofarm (Pabianice, Poland)	tablet	600	2.18	0.25
30	Nettle herb extract (*Urtica dioica* L.)	Extract of green tea leaves, Cayenne pepper fruit, Indian nettle root, green coffee seeds	Olimp Labs (Dębica, Poland)	tablet	675	0.56	0.04
31	Horsetail herb extract (*Equisetum arvense* L.)	Hops cone extract, glucomannan, spirulina, aloe vera leaf juice extract	Noble Health (Warsaw, Poland)	tablet	1000	3.78	0.20
32	Spirulina (*Arthrospira platensis*)	-	Swanson (Fargo, ND, USA)	tablet	500	4.43	0.23
33	Spirulina (*Arthrospira platensis*)	Chlorella	Noble Health (Warsaw, Poland)	tablet	800	0.62	0.13
34	Spirulina (*Arthrospira platensis*)	-	Oleofarm (Pietrzykowice, Poland)	powder within the capsule	411	10.25	0.07
35	Spirulina (*Arthrospira platensis*)	Extract of bitter orange fruit, prickly pear fruit, Indian barberry, artichoke, horsetail, nettle, cinnamon	Colfarm (Mielec, Poland)	tablet	750	4.40	0.06
36	Spirulina (*Arthrospira platensis*)	-	Ginseng Poland (Kalisz, Poland)	tablet	500	14.65	1.64
37	Spirulina (*Arthrospira platensis*)	-	Ginseng Poland (Kalisz, Poland)	tablet	500	6.68	1.03
38	Spirulina (*Arthrospira platensis*)	-	My Life (Gniezno, Poland)	tablet	3000	1.89	0.06
39	Green coffee bean extract (*Coffea arabica* L.)	Guarana seed extract	Olimp Labs (Dębica, Poland)	powder within the capsule	785	0.73	0.35
40	Chlorella (*Chlorella vulgaris*)	Extract of green tea leaves, bitter orange fruit, Cayenne pepper fruit, chromium	Noble Health (Warsaw, Poland)	tablet	1000	2.44	0.28
41	Green coffee bean extract (*Coffea arabica* L.)	Extract from bean seeds, green tea leaves, bitter orange fruit, prickly pear fruit	Colfarm (Mielec, Poland)	tablet	966	3.14	0.29
42	Malabar tamarind fruit extract (*Garcinia Cambogia*)	-	Natur Produkt Pharma	powder within the capsule	650	2.53	0.29
43	Young barley (*Hordeum vulgare* L.)	-	Avet Pharma (Warsaw, Poland)	tablet	350	0.55	0.03
44	Young barley (*Hordeum vulgare* L.)	-	Healthways (Warsaw, Poland)	tablet	620	3.84	0.33
45	White mulberry leaf extract (*Morus alba* L.)	*Gymnema sylvestre* leaf extract	Starpharma (Lesznowola, Poland)	tablet	700	12.02	1.04
46	Green tea extract (*Camellia sinensis* L.)	-	Virde (Chorzów, Poland)	powder within the capsule	220	18.36	1.34
47	Chlorella	-	Green Ways (Rybnik, Poland)	tablet	330	18.36	2.96

**Table 2:** Statistical analysis of the Hg content of weight loss-supporting DSs tested [µg/kg].

Main Active Ingredient	N	AM ± SD	Me	Min	Max	Quartiles		CV [%]
						Q_1_	Q_3_	
spirulina	7	6.13 ± 4.90	4.43	0.62	14.65	1.89	10.25	80
white mulberry	6	10.98 ± 10.00	8.11	2.15	28.74	4.16	14.63	91
young barley	6	5.09 ± 4.13	4.59	0.55	10.89	1.13	8.79	81
Malabar tamarind	4	3.99 ± 3.69	2.93	0.80	9.29	1.66	6.32	92
chlorella	3	21.58 ± 23.24	18.36	0.12	46.27	0.12	46.27	108
green coffee	3	2.10 ± 1.24	2.44	0.73	3.14	0.73	3.14	59
l-carnitine	3	1.07 ± 0.27	1.16	0.77	1.28	0.77	1.28	25
African mango	3	1.57 ± 0.64	1.33	1.09	2.31	1.09	2.31	41
all	47	5.80 ± 8.47	2.44	0.12	46.27	1.09	6.68	146

N—number of samples, AM—arithmetic mean, SD—standard deviation, Me—median, Min—minimum, Max—maximum, Q_1_—first quartile, Q_3_—third quartile, CV—coefficient of variation.

**Table 3:** Statistical analysis of the Hg content of DSs tested according to form [µg/kg].

Form	N	AM	SD	Me	Min	Max
powder within capsule	17	7.15	11.48	1.39	0.12	46.27
tablet	30	5.03	6.29	2.79	0.22	28.74

N—number of samples, AM—arithmetic mean, SD—standard deviation, Me—median, Min—minimum, Max—maximum, *p* > 0.05.

**Table 4:** Health risk assessment indices for the tested weight loss-supporting DSs.

No.	EDI	EWI	TWI%_inorganic mercury_	TWI%_methylmercury_	THQ
1	0.0007	0.0046	0.002	0.005	0.09
2	0.0006	0.0039	0.001	0.004	0.08
3	0.0763	0.5344	0.191	0.587	10.91
4	0.0005	0.0038	0.001	0.004	0.08
5	0.0005	0.0038	0.001	0.004	0.08
6	0.0015	0.0107	0.004	0.012	0.22
7	0.0003	0.0022	0.001	0.002	0.05
8	0.0070	0.0488	0.017	0.054	1.00
9	0.0020	0.0140	0.005	0.015	0.29
10	0.0036	0.0251	0.009	0.028	0.51
11	0.0001	0.0006	0.000	0.001	0.01
12	0.0011	0.0078	0.003	0.009	0.16
13	0.0024	0.0167	0.006	0.018	0.34
14	0.0044	0.0308	0.011	0.034	0.63
15	0.0024	0.0171	0.006	0.019	0.35
16	0.0083	0.0581	0.021	0.064	1.19
17	0.0007	0.0047	0.002	0.005	0.10
18	0.0006	0.0041	0.001	0.005	0.08
19	0.0048	0.0335	0.012	0.037	0.68
20	0.0053	0.0374	0.013	0.041	0.76
21	0.0006	0.0040	0.001	0.004	0.08
22	0.0066	0.0462	0.016	0.051	0.94
23	0.0603	0.4224	0.151	0.464	8.62
24	0.0025	0.0177	0.006	0.019	0.36
25	0.0069	0.0481	0.017	0.053	0.98
26	0.0013	0.0090	0.003	0.010	0.18
27	0.0026	0.0180	0.006	0.020	0.37
28	0.0020	0.0139	0.005	0.015	0.28
29	0.0026	0.0183	0.007	0.020	0.37
30	0.0008	0.0053	0.002	0.006	0.11
31	0.0038	0.0264	0.009	0.029	0.54
32	0.0044	0.0310	0.011	0.034	0.63
33	0.0030	0.0210	0.007	0.023	0.43
34	0.0126	0.0884	0.032	0.097	1.80
35	0.0033	0.0231	0.008	0.025	0.47
36	0.0439	0.3076	0.110	0.338	6.28
37	0.0201	0.1404	0.050	0.154	2.86
38	0.0340	0.2380	0.085	0.262	4.86
39	0.0011	0.0080	0.003	0.009	0.16
40	0.0049	0.0342	0.012	0.038	0.70
41	0.0030	0.0213	0.008	0.023	0.43
42	0.0033	0.0230	0.008	0.025	0.47
43	0.0004	0.0027	0.001	0.003	0.05
44	0.0048	0.0333	0.012	0.037	0.68
45	0.0168	0.1178	0.042	0.129	2.40
46	0.0081	0.0565	0.020	0.062	1.15
47	0.1212	0.8481	0.303	0.932	17.31

EDI—estimated daily intake, EWI—estimated weekly intake, TWI—tolerable weekly intake, %TWI_inorganic mercury_, %TWI_methylmercury_, THQ—the Target Hazard Quotient.

## Data Availability

The original contributions presented in this study are included in the article. Further inquiries can be directed to the corresponding author.
